# Single florescent nanodiamond in a three dimensional ABEL trap

**DOI:** 10.1038/srep16669

**Published:** 2015-11-12

**Authors:** Metin Kayci, Aleksandra Radenovic

**Affiliations:** 1 Institute of Bioengineering, Ecole Polytechnique Federale de Lausanne (EPFL) CH -1015 Lausanne, Switzerland

## Abstract

Three dimensional single particle trapping and manipulation is an outstanding challenge in various fields ranging from basic physics to life sciences. By monitoring the response of a trapped particle to a designed environment one can extract its characteristics. In addition, quantum dynamics of a spatially scanned well-known particle can provide environmental information. Precise tracking and positioning of such a particle in aqueous environment is crucial task for achieving nano-scale resolution. Here we experimentally demonstrate three dimensional ABEL trap operating at high frequency by employing a hybrid approach in particle tracking. The particle location in the transverse plane is detected via a scanning laser beam while the axial position is determined by defocused imaging. The scanning of the trapped particle is accomplished through a nano positioning stage integrated to the trap platform.

Single particle trapping and manipulation can provide inner dynamics of single molecules that are not resolvable in ensemble level averaging measurements. Optical trap is one of the most powerful techniques that has been used for nano-scale positioning in biophysics and quantum optics. Recently, it has also been proposed as three dimensional scanning tool for nitrogen vacancy embedded single nanocrystal employed in quantum sensing^1^. However, this technique is not practical for particles smaller in size since the optic forces required for stable 3D trapping are proportional to the particle’s volume. High optic powers on such particles may introduce heating that could perturb the biological environment^2^. Moreover, as any particles near the beam focus are subjected to the trapping forces it is not selective. Therefore, single particle level trapping requires extremely low concentration which is not the case for very crowded physiological environments. Instead, electromagnetic tweezers^3^ can provide single particle selectivity since they operate by active feedback control. However the trapping is limited to magnetic beads and high driving currents on electromagnets could result in a substantial heating which in turn requires an active cooling system. Less common, modalities such as dielectrophoresis^4^, acoustophoresis^5^, Paul trapping^6^ have been also used in single particle experiments, but unfortunately all these techniques have constraints either related to the trapping environment or trapped particle characteristics.

ABEL trap is a promising technique that can overcome size limitations as well as the environmental issues. It combines a very fast detection scheme with a real time feedback compensating the Brownian motion. Unlike optical or magnetic tweezers, the electo-kinetic forces applied in ABEL trap scale linearly with the particle’s radius. Single dye molecules of sub-nanometer size trapping has been achieved with a very low optical excitation used in the position tracking^7^. Given that the Brownian trajectories of multiple particles in the trap area are uncorrelated; hence only one is subjected to the correct feedback, exactly one is trapped during this process. Also, as all particles are exposed to the same feedback the electro-kinetic force on the environment is non-perturbative. The instant feedback doesn’t generate a potential well in the trap area but a uniform field directing the particle. This also avoids any clustering and agglomeration that occur in passive trapping approaches when working in a dense dispersions. Providing both electroosmotic and electrophoretic actuations the trapping is not limited by dielectric, magnetic or charge properties of the particle. Therefore, any traceable particle can be trapped.

Although a two dimensional ABEL trap can provide precise measurements without physical perturbations like surface tethering^8^ or excessive heating^2^ the confinement in axial dimension by the geometry of trapping cavity may introduce undesired interactions. As the cavity depth used for confinement is very small and nano-scale particles have very high diffusion coefficient, during a trapping experiment such particles will have very high surface collision rate. For instance, Rhodamine B in a cavity of 800 nm depth collides to the wall with 87 kHz frequency^9^. This interference may induce florescence quenching or adsorption of the particle limiting the trap period and measurement precision. Improvement on microfluidics material and modification of the surface chemistry^10^ has been proposed to remove such effects but it requires complex fabrication process and has high cost drawback^11^. Alternatively, a three dimensional ABEL trap can provide collision free manipulation without engineering of the chemical and the geometric properties of the cavity. Indeed, a recent work has utilized a sets of micro electrodes patterned on two fused silica layers forming the trap chamber to achieve three dimensional control over the particle monitored with a CCD camera^12^. However, the close configuration of the electrodes in the trap chamber degrades the trap performance when a contamination process screens the applied voltages. Also, video based position detection employed for all dimensions extends the feedback latency that plays a critical role in the trap resolution. Here we demonstrate soft lithography based microfluidics exploiting both electroosmotic and electrophoretic forces through electrodes inserted to the ports distant to the trapping chamber. To preserve high feedback frequency achieved in conventional laser guided 2D ABEL trap, we employed laser guided in-plane transverse detection scheme, while independently running image processing that estimates the axial position. With here presented approach, we extend the operating environment of the nitrogen vacancy (NV) defects hosted in nanodiamonds from 2D to 3D opening the possibility to use for NV based sensing and imaging applications in physiological environments.

## Microfluidic cell

Connected microchannels forming the microfluidics provide the interface between the physical voltages on the electrodes and the trap chamber. The forces introduced by the microfluidics are the main control units correcting the position of the particle. In our 3D microfluidics trap both electrophoretic and electroosmotic forces are contributing to the electro-kinetic mobility due to the converging electric field on the trapped particle and long micro channels in which mobile ions are generating bulk flow.

**Figure 1:**
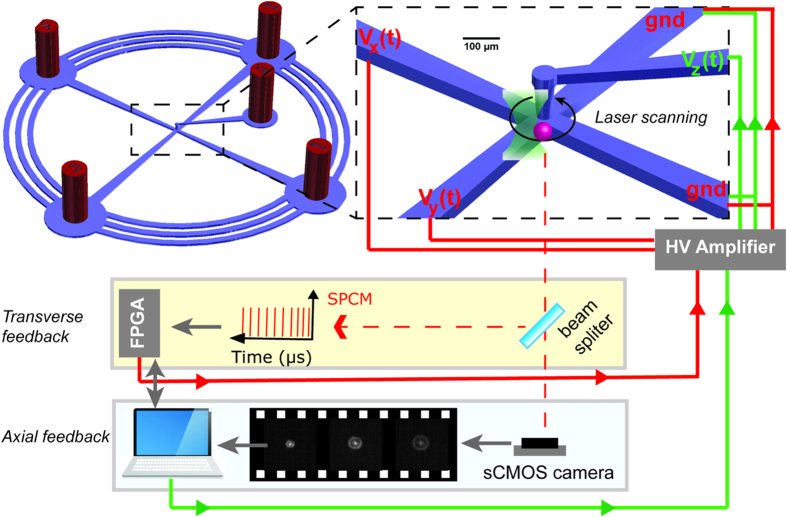
The schematics representing the microfluidics geometry. Several outer rings are connecting the ports to suppress the undesired drifts in the channels. The trap chamber in the center connected to three orthogonal channels each for one dimensional control. The feedback voltages on the electrodes (v_x_, v_y_, v_z_) generate the electric field actuating electrophoresis and electroosmotic forces. The ports are also functioning as inlet and outlet.

The microfluidics in PDMS (polydimethylsiloxane) providing the transverse plane position control was fabricated using multilayer SU8 masters (for details see Supporting Information). The trap chamber in the center was formed by precisely punching the intersection of the channels each responsible for one dimensional control. Then, a second microfluidic layer was cascaded in such way that a new channel connected to the chamber introduces the position control in the axial dimension (see Supporting Information). Besides the simple integration of insertable electrodes this configuration also provides the control over the full axial chamber size that is very helpful in suppressing the background florescence noise (Fig. 1).

## Three Dimensional Position Tracking

**Figure 2:**
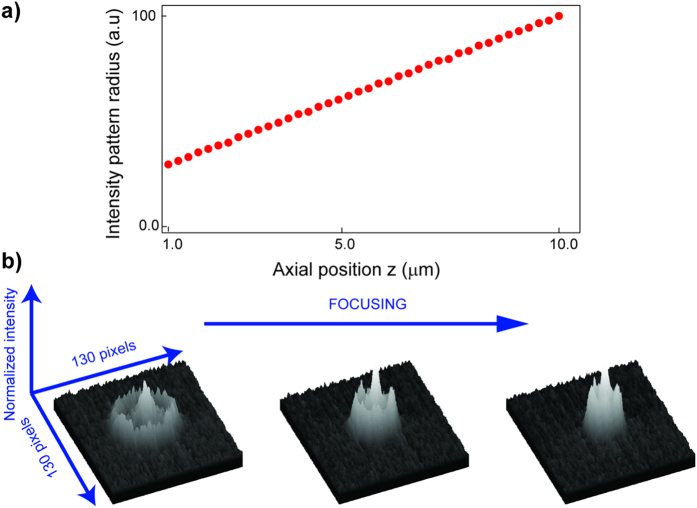
(**a**) The size of the outermost ring in the intensity pattern of FND scales linearly with the distance to the focal plane, z. (**b**) Surface profiles of the grayscale images visualizing the intensity patterns at three different axial locations. Defocusing stretches the pattern, enlarges the outermost ring size and degrades the image contrast.

The axial position of the particle was estimated through defocused imaging proposed by *Speidel et al.*^13^. The idea is to detect the off-focus distance that is encoded into the intensity pattern in the image plane. It is shown that for a point-like particle the size of the outermost ring of the pattern scales linearly with the axial position. Here, we demonstrated the relation for a wide defocusing range starting from ≈1 μm above the focus plane (Fig. 2). Since the random walk of a particle takes a time-bin of *t* = *δ*^2^/2*D*, where *D* is the diffusion coefficient, this range also defines the maximum feedback latency tolerated in a stable trapping. The optimal target position is chosen such that it extends the path particle can travel before it escapes. Once the position determined the feedback is applied to the corresponding fluidic channel to compensate the Brownian motion induced offset.

While it has sub nanometer detection precision in the axial dimension this detection scheme doesn’t provide high spatial resolution in transverse plane. A large defocus value leads a spread in the intensity pattern and a consequent degrade in the image contrast where the location with the maximum intensity value defines the particle position in the transverse plane. Also, since the image processing for all dimensions extends the feedback latency an independently running detection scheme is preferable for high temporal resolution. Therefore, the detection in the transverse plane was performed using a real time *Kalman filter* implemented on a field programmable gate array (FPGA) device and a rotating laser beam around the target point that excites the particle with a uniform profile as reported in *Kayci et al.*^14^. Using an avalanche photo diode (APD) that feeds the filter on the FPGA device the positon vector can be precisely determined upon single photon arrivals. The correcting filter gain adjusting the feedback strength is determined by instant photon count rate and the geometry of the scanning beam (see Supporting Information). Similar to the axial trapping, to keep the particle at the target location any shifts in the transverse plane is cancelled through the in-plane fluidic channels. While for the axial dimension the feedback rate is mainly limited by image processing speed (≈4 ms) and the camera exposure time (≈10 ms) for the transverse plane it is a function of photon counts on the APD and the frequency of the scanning laser beam.

## Florescent Nanodiamond Trap

To test the performance of here presented three dimensional ABEL trap we have used single florescence nano diamonds (FNDs) due to their non-blinking and non-bleaching properties. Thanks to its optic and spin properties NV embedded in a single FND has received a remarkable interest in the last decade. It has been validated as nanoscale quantum sensor for physical parameters ranging from temperature^15^, pressure^16^, and ion concentrations^17^ to external electric and magnetic field^18^,^19^. As the particle presents no bleaching or blinking behavior under ambient conditions it is well-matched to the ABEL trap manipulation approach. More recently, we showed such FNDs can be used as sensitive magnetometer in two dimensional ABEL trap operating in fluidics^14^. After the successful realization of the hybrid detection scheme in the tracking here we verified the performance of technique by three dimensional trapping and scanning of 25 nm sized single FNDs.

**Figure 3:**
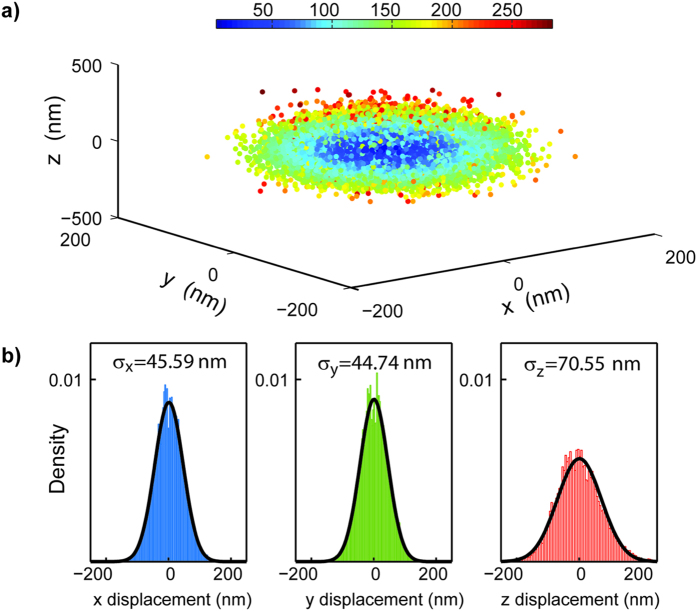
(**a**) The estimated positions at which the FND detected during a trap event. The color bar visualizes the distance to the target point (**b**) x, y, z displacement histograms during the trap event. The confinement in the transverse plane (x, y) is better than the axial dimension due to the faster detection scheme.

**Figure 4:**
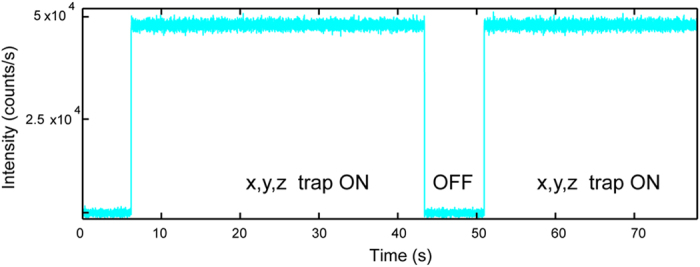
The photon counts on the APD when the trap state switches. The profile verifies non-photo bleaching and non-blinking behavior of the FND. The high contrast in the profile demonstrates the feasibility of sensing and detection applications exploiting florescence decay at magnetic resonance. The intensity of the trapped FND remains constant in the whole axial range corresponding to 10 μm when FND is scanned with the nano-positioning stage. Current design of microfluidic chip limits the axial range to 10 μm.

The particle was confined to a nano-scale volume with the displacement values of *σ*_*x*_ = 45.59 nm, *σ*_*y*_ = 44.74 nm and *σ*_*z*_ = 70.55 nm setting the trap stiffness (Fig. 3). As the uniform excitation is performed by a rotating pencil like beam with high depth of focus and the particle is not photo bleaching or blinking the emission follows a digital profile when the trap sate switches (Fig. 4). Three dimensional scan of the relative position in the buffer was performed using a nano positioning stage. Basically the displacement of the stage was conceived as perturbation to be cancelled by the electro kinetic forces as done for the fluctuations driven by Brownian motion.

## Conclusion

Combining computationally-independent detection schemes, scanning laser beam guided position estimation and defocused imaging, high frequency three dimensional particle tracking is demonstrated. The method is verified by ABEL trapping of a single 25 nm sized FND containing NV defects diffusing in fluidics. The microfluidic cell is fabricated through the soft lithography process of PDMS. Extension of the ABEL trapping into the third dimension facilitates the scanning of the trapped particle in the fluidic volume where the temporal and the spatial scanning resolutions are limited by the nano positioning stage. This provides a remarkable sensing tool to map the three dimensional distribution of the physical quantities in a fluidic environment.

## Additional Information

**How to cite this article**: Kayci, M. and Radenovic, A. Single florescent nanodiamond in a three dimensional ABEL trap. *Sci. Rep.* **5**, 16669; doi: 10.1038/srep16669 (2015).

## Supplementary Material

Supplementary Information

## References

[b1] Geiselmann M. *et al.* Three-dimensional optical manipulation of a single electron spin. Nature Nanotechnology 8, 175–179 (2013). 10.1038/nnano.2012.259 23396312

[b2] Peterman E. J., Gittes F. & Schmidt C. F. Laser-induced heating in optical traps. Biophysical journal 84, 1308–1316 (2003). 12547811 10.1016/S0006-3495(03)74946-7 PMC1302707

[b3] Gosse C. & Croquette V. Magnetic tweezers: micromanipulation and force measurement at the molecular level. Biophysical journal 82, 3314–3329 (2002). 12023254 10.1016/S0006-3495(02)75672-5 PMC1302119

[b4] Wu M. C. Optoelectronic tweezers. Nature Photonics 5, 322–324 (2011).

[b5] Tran S. B. Q., Marmottant P. & Thibault P. Fast acoustic tweezers for the two-dimensional manipulation of individual particles in microfluidic channels. Applied Physics Letters 101, Artn 114103 (2012).

[b6] Guan W. H., Joseph S., Park J. H., Krstic P. S. & Reed M. A. Paul trapping of charged particles in aqueous solution. Proc. Natl Acad. Sci. USA 108, 9326–9330 (2011). 21606331 10.1073/pnas.1100977108 PMC3111334

[b7] Fields A. P. & Cohen A. E. Electrokinetic trapping at the one nanometer limit. Proc. Natl Acad. Sci. USA 108, 8937–8942 (2011). 21562206 10.1073/pnas.1103554108 PMC3107292

[b8] Zhuang Z. Y., Jewett A. I., Soto P. & Shea J. E. The effect of surface tethering on the folding of the src-SH3 protein domain. Phys Biol 6, 015004 (2009). 19208934 10.1088/1478-3975/6/1/015004

[b9] King J. K. Three-Dimensional Electrokinetic Trapping of a Single Fluorescent Nanoparticle in Solution (2013).

[b10] Bockenhauer S. D. & Moerner W. E. Photo-induced conformational flexibility in single solution-phase peridinin-chlorophyll-proteins. J Phys Chem A 117, 8399–8406 (2013). 23919352 10.1021/jp405790a

[b11] Cohen A. E. & Moerner W. E. An All-Glass Microfluidic Cell for the Abel Trap: Fabrication and Modeling. Proceedings of SPIE 5930, 59300S-59300S-8, 2005.

[b12] King J. K., Canfield B. K. & Davis L. M. Three-dimensional anti-Brownian electrokinetic trapping of a single nanoparticle in solution. Applied Physics Letters 103, 043102 (2013).

[b13] Speidel M., Jonas A. & Florin E. L. Three-dimensional tracking of fluorescent nanoparticles with subnanometer precision by use of off-focus imaging. Opt Lett 28, 69–71 (2003). 12656488 10.1364/ol.28.000069

[b14] Kayci M., Chang H. C. & Radenovic A. Electron Spin Resonance of Nitrogen-Vacancy Defects Embedded in Single Nanodiamonds in an ABEL Trap. Nano Lett 14, 5335–5341 (2014). 25111386 10.1021/nl5023964

[b15] Kucsko G. *et al.* Nanometre-scale thermometry in a living cell. Nature 500, 54–U71 (2013). 23903748 10.1038/nature12373 PMC4221854

[b16] Doherty M. W. *et al.* Electronic Properties and Metrology Applications of the Diamond NV- Center under Pressure. Phys Rev Lett 112, Artn 047601 (2014). 10.1103/PhysRevLett.112.047601 24580492

[b17] Steinert S. *et al.* Magnetic spin imaging under ambient conditions with sub-cellular resolution. Nature Communication 4, Artn 1607 (2013). 10.1038/ncomms2588 23511472

[b18] Dolde F. *et al.* Electric-field sensing using single diamond spins. Nature Physics 7, 459–463 (2011).

[b19] Maze J. R. *et al.* Nanoscale magnetic sensing with an individual electronic spin in diamond. Nature 455, 644–647 (2008). 18833275 10.1038/nature07279

