# Supplementary material for: Single florescent nanodiamond in a three dimensional ABEL trap
Source: Sci Rep. 2015 Nov 12;5:16669. doi: 10.1038/srep16669 (PMC4642320; doi:10.1038/srep16669)
Supplement: Supplementary Information [file srep16669-s1.pdf]

Supporting Information

Single florescent nanodiamond in a three dimensional ABEL trap

Metin Kayci, Aleksandra Radenovic*[1]

[1]*Institute of Bioengineering , Ecole Polytechnique Federale de Lausanne (EPFL) CH -1015 Lausanne, Switzerland*

**Correspondence should be addressed to::metin.kayci@epfl.ch and aleksandra.radenovic@epfl.ch*

Table of contents

1. Image processing based position estimation for axial dimension
2. Kalman-filter based position estimation in the transverse plane
3. Details on microfluidic cell fabrication
4. **SI Figure 1.** Selected image frames obtained using sCMOS camera together with the corresponding binary transformation used to estimate particle axial position
5. **SI Figure 2.** Photograph of the transverse plane microfluidics formed in a thin PDMS layer
6. **SI Figure 3.** Bight-field optical micrographs of the layers the microfluidics assembly
7. **SI Figure 4.** Photograph of the three dimensional ABEL trap microfluidic chip

Image processing based position estimation for axial dimension

The images are acquired through a fast CMOS (Andor Neo) camera streaming the data into the computer. The region of interest is set to the maximum size that the intensity pattern of the target particle can reach during the active feedback intervals. Image processing is performed on NI Vision Module environment. A threshold suppressing the background noise is applied to the luminance data extracted from the image frames. This process also filters any particle with the intensity value falling outside of the threshold range hence providing selectivity. After subsequent *dilute, erode* operations with the calibrated parameters the size of the outmost ring in the intensity pattern is detected through processed image in binary (**Fig. 1**).

Kalman-filter based position estimation in the transverse plane

In this approach, a pattern is fed into Electro-Optic Deflectors scanning the sample plane. Then through florescence photons arrival times (detected by a Single Photon Counting Module SPCM) the position of the molecule is obtained. As the beam has finite size, these positions are not precise and so called measured positions with a measurement noise. A digital filter, Kalman fpilter, is used to estimate the real positions. A summary of the recursive algorithm is shown below (the discussion is restricted to 1D). The filter is implemented in a NI-FPGA device). Details on the setup can be found in Kayci *et al*.[1].

Predict step:

$$x_n^- = x_{n-1} + \mu.\Delta t.v$$

$$p_n^- = p_{n-1} + 2D.\Delta t$$

Update step:

$$x_n = x_n^-.(1-\gamma) + \gamma.x_{measured}$$

$$p_n = p_n^-.(1-\gamma)$$

x_n^- and p_n^- are the prior estimates for the mean and variance of the position respectively. x_n and p_n are the posterior estimates. D is the diffusion constant, v is the physical voltage applied to the electrodes, μ is the electro-kinetic mobility and Δt is the time interval between the update steps. $x_{measured}$ is the measured position defined by the instant position of the scanning laser beam. The posterior variance is minimized through the parameter called Kalman-gain, $\gamma = p_n^-.(p_n^- + w/n)^{-1}$ where w refers to the measurement noise due to the scanning beam geometry and n refers to the instant photon counts.

Microfluidic cell fabrication

The SU8 master defining the geometry of the transverse plane microfluidics was fabricated in many layers form such that the thickness of the channels is minimum around the trap chamber. This facilitates better electrical contact for the electrodes and higher electro kinetic mobility in the trap chamber. The microfluidics were formed using PDMS spin coating of the master with a thickness value setting the depth of the trap chamber. Then, the mold was peeled off and transferred to a clean PDMS base for punching process (**Fig. 2**). The location of the trap center was punched as circular cavity of 300 µm in size to connect the fluidic channel in a second layer. Similarly, the fluidic channel in the second layer was made and plasma bonded to the first one such that the end port of the channel encloses the trap chamber (**Fig. 3**). This connection couples the control into the axial dimension. The alignment was performed precisely under a microscope. Then the cell was punched to introduce the integration ports for electrodes. These ports also serve as inlet and outlet for the sample. Finally, the microfluidic device was plasma treated for hydrophilic property and assembled by the integration of glass cover slip (**Fig. 4**).

References

1 Kayci, M., Chang, H.-C. & Radenovic, A. Electron spin resonance of nitrogen-vacancy defects embedded in single nanodiamonds in an ABEL trap. *Nano letters* **14**, 5335-5341 (2014).

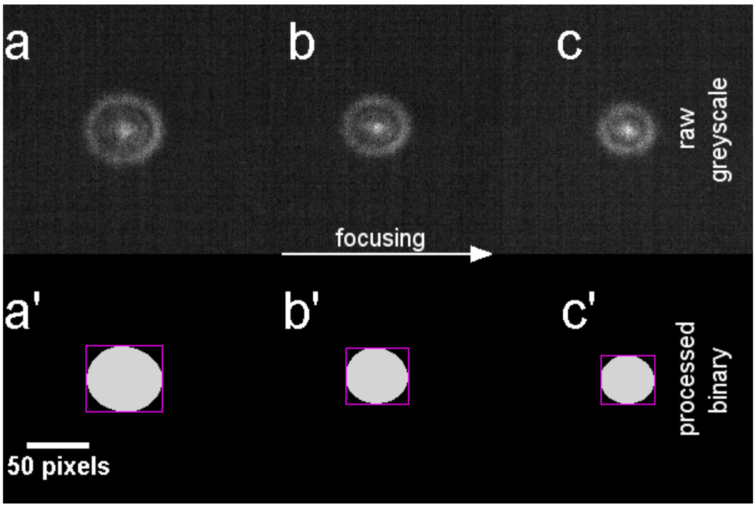

SI Figure 1. Selected image frames obtained using sCMOS camera. Simple and fast image analysis extracts the particle axial positions based on the estimation of the size of the outer most ring in the intensity pattern (a, b, c). The results of the image processing are displayed in (a’, b’, c’) for the corresponding frames.

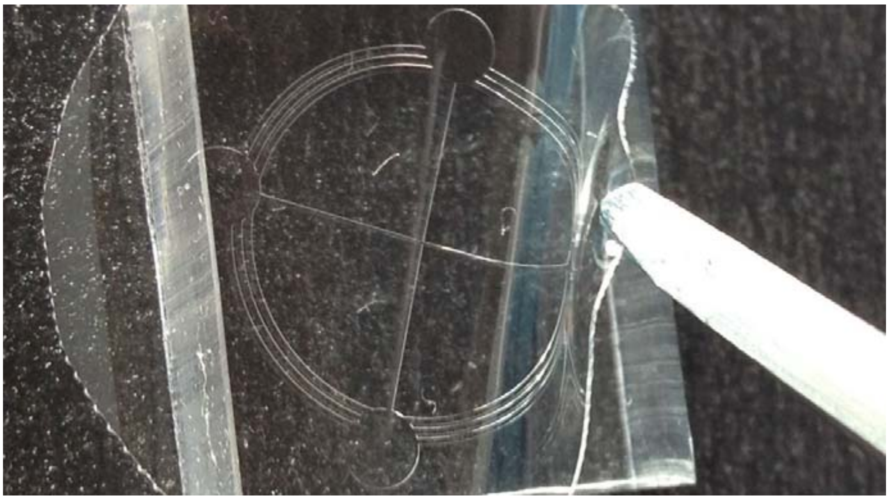

SI Figure 2. Photograph of the transverse plane microfluidics formed in a thin PDMS layer. The layer is transferred to a thicker one for punching process defining the trap chamber.

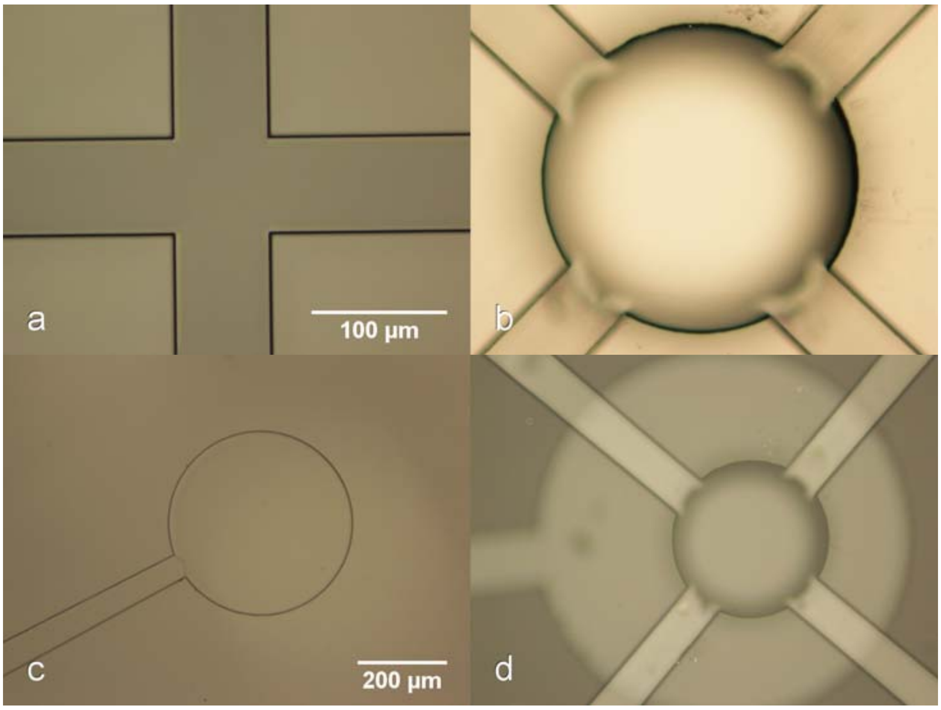

SI Figure 3. Bight-field optical micrographs of the layers the microfluidics assembly. **(a)** The microfluidic channels formed in the first layer to be used in the transverse plane position control. **(b)** The trap chamber is formed at the intersection of the channels using a punch tool. **(c)** A single channel in a second layer is formed to be used in the axial position control. **(d)** Two layers of the microfluidics are cascaded to introduce 3D position control for the particle diffusing in the trap chamber.

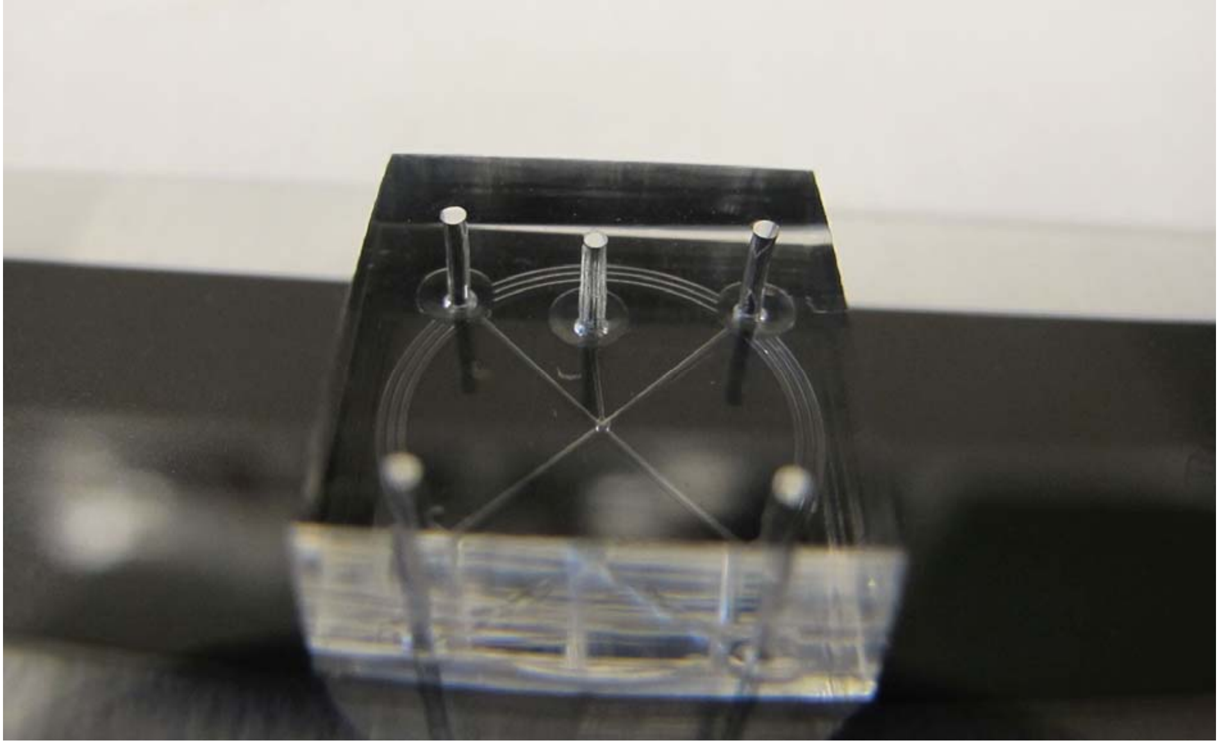

SI Figure 4. Photograph of the three dimensional ABEL trap microfluidic chip. The ports are punched for the integration of the electrodes and loading the sample.
